# Effect of Electrospun Fiber Mat Thickness and Support Method on Cell Morphology

**DOI:** 10.3390/nano9040644

**Published:** 2019-04-20

**Authors:** Mark A. Calhoun, Sadiyah Sabah Chowdhury, Mark Tyler Nelson, John J. Lannutti, Rebecca B. Dupaix, Jessica O. Winter

**Affiliations:** 1 Department of Biomedical Engineering, The Ohio State University, Columbus, OH 43210, USA; Calhoun.89@osu.edu (M.A.C.); Nelsonmt05@gmail.com (M.T.N.); 2 Department of Mechanical and Aerospace Engineering, The Ohio State University, Columbus, OH 43210, USA; Chowdhury.68@osu.edu (S.S.C.); Dupaix.1@osu.edu (R.B.D.); 3 Department of Materials Science and Engineering, The Ohio State University, Columbus, OH 43210, USA; Lannutti.1@osu.edu; 4 William G. Lowrie Department of Chemical and Biomolecular Engineering, The Ohio State University, 453 CBEC, 151 W. Woodruff Ave., Columbus, OH 43210, USA

**Keywords:** electrospun fiber mats, mechanobiology, glioblastoma, biomaterials, finite element modeling

## Abstract

Electrospun fiber mats (EFMs) are highly versatile biomaterials used in a myriad of biomedical applications. Whereas some facets of EFMs are well studied and can be highly tuned (e.g., pore size, fiber diameter, etc.), other features are under characterized. For example, although substrate mechanics have been explored by several groups, most studies rely on Young’s modulus alone as a characterization variable. The influence of fiber mat thickness and the effect of supports are variables that are often not considered when evaluating cell-mechanical response. To assay the role of these features in EFM scaffold design and to improve understanding of scaffold mechanical properties, we designed EFM scaffolds with varying thickness (50–200 µm) and supporting methodologies. EFM scaffolds were comprised of polycaprolactone and were either electrospun directly onto a support, suspended across an annulus (3 or 10 mm inner diameter), or “tension-released” and then suspended across an annulus. Then, single cell spreading (i.e., Feret diameter) was measured in the presence of these different features. Cells were sensitive to EFM thickness and suspended gap diameter. Overall, cell spreading was greatest for 50 µm thick EFMs suspended over a 3 mm gap, which was the smallest thickness and gap investigated. These results are counterintuitive to conventional understanding in mechanobiology, which suggests that stiffer materials, such as thicker, supported EFMs, should elicit greater cell polarization. Additional experiments with 50 µm thick EFMs on polystyrene and polydimethylsiloxane (PDMS) supports demonstrated that cells can “feel” the support underlying the EFM if it is rigid, similar to previous results in hydrogels. These results also suggest that EFM curvature may play a role in cell response, separate from Young’s modulus, possibly because of internal tension generated. These parameters are not often considered in EFM design and could improve scaffold performance and ultimately patient outcomes.

## 1. Introduction

Electrospun fibers are used widely across a range of applications, including filtration [1], drug delivery [2] and tissue engineering [3]. This is a result of the high degree of tunability of their pore size [4], fiber diameter [5] and degradation rate [6]. Generally the most important aspect of electrospun fiber mats (EFMs) as biomedical scaffolds is their fibrous topography [7], which influences cell morphology [8], migration [9] and gene regulation [10]. This has enabled their use in vascular grafts [11], organ replacement [12] and cancer treatment [13]. Thus, EFM features, such as fiber diameter and pore size features [14,15,16] have been well studied. Further, many studies address the mechanics of EFMs, which are powerful regulators of cell phenotype [17,18]. However, few of these go beyond Young’s modulus, which assumes linear elasticity that may be inconsistent with the polymers used in EFM scaffolds.

Further, EFMs can be presented in a variety of conformations that may alter their mechanical properties. For example, EFMs are often synthesized on the surface of a much stiffer solid support, such as tissue culture polystyrene [19]. These mechanical nuances could potentially have a large effect on observed cell response, as we and others have shown the presence of edge effects in similar hydrogel culture models supported on glass or polystyrene that influence cell morphology [20,21]. Additionally, EFMs may be synthesized across an annular gap with support on the outer edges only (i.e., suspended). Such a configuration increases internal tension in the fibers as the EFM curvature increases. The effects of this configuration have not been widely explored. Addressing mechanical nuances in EFM scaffolds represents a valuable opportunity to advance understanding and to enable the design of next generation of EFM biomaterials.

Here, we employed EFMs in different scaffold configurations to correlate features of the mechanical environment to changes in cell morphology, extending our studies beyond Young’s modulus. In particular, similar to our previous study in hydrogels [20], we examined cell morphology as a function of interfacial mechanics by altering the EFM support material to determine if cells cultured on EFM supports can “feel” the underlying substrate. Cell morphology often relates to or precedes other cell behavior in a myriad of conditions [17,22,23,24], and thus is a critical characteristic reporter of cell behaviors. We also evaluated the effect of EFM presentation: such as suspension across a gap, which induces curvature and may increase deformability versus support on a solid material; and we investigated the effect of releasing EFM internal residual tension that occurs during the spinning process, which would also alter deformability, alignment and presentation of focal adhesion sites. In the latter cases, the EFM material stiffness, as measured by Young’s modulus, remains relatively constant; however, deformability of the fibers is altered, permitting subtle mechanical effects to be observed. As a model system, glioblastoma cells were employed because of their highly invasive nature and dysregulation in cell signaling related to migration and morphology. These studies highlight the importance of considering factors beyond Young’s modulus in materials design to more fully understand the interaction between substrate mechanics and cell response.

## 2. Materials and Methods

### 2.1. Preparation of Aligned Polycaprolactone Electrospun Fiber Mat Constructs

**Figure 1:**
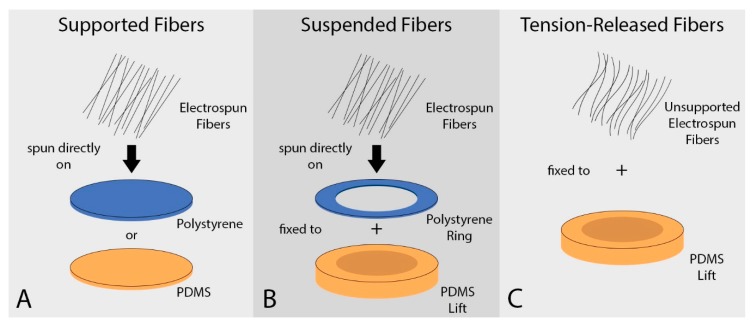
Schematic of Electrospun Fiber Mats. Electrospun fiber mats (EFMs) were either spun directly onto a stiff support (**A**), spun across a gap (**B**), or spun unto foil, removed, and then fixed across a gap (**C**).

Aligned polycaprolactone (PCL) fiber mats were prepared by electrospinning onto a rotating mandrel, as described previously [25]. Briefly, 5 wt% PCL (Mn 70,000–90,000, Sigma-Aldrich, St. Louis, MO, USA) solution in 1,1,1,3,3,3-hexafluoro-2-propanol (HFP) (>99% purity; Oakwood Products, Inc., Columbia, SC, USA) was electrospun at 4 mL/h at a 20 cm needle-to-collector distance [26]. The rotating mandrel was set to maintain a linear velocity at the collecting surface of 15 m/s. Fiber mat thickness was varied from 50, 100 and 200 microns by spinning for ~45 min, ~1 h 30 min and ~4 h, respectively. Fiber mats were spun either directly onto a supporting material or across an annular gap (Figure 1). Fiber mats were rendered hydrophilic for cell culture by air plasma treatment (Harrick Plasma, Ithaca, NY, USA) under vacuum at ~700 mTorr and with a plasma radio frequency of 8–12 MHz for 3 min. Fiber mats were used immediately in experiments.

#### 2.1.1. Polydimethylsiloxane- and Polystyrene-Supported Fiber Mats

Supported fiber mats (Figure 1A) were synthesized by fixing polydimethylsiloxane (PDMS) and polystyrene (PS) wafers to the rotating mandrel with double sided tape. PS wafers (Multi-Plastics Inc., Lewis Center, OH, USA) were 0.2 mm thick and cut to a 10 mm diameter with an arch punch (Grainger, Columbus, OH, USA). PDMS was made from a combination of SylGard 184 and SylGard 527 (Dow Corning, Midland, MI, USA) to vary stiffness without changing surface charge or chemistry [27]. Stiffer “PDMS 100/0” was made of 100% SylGard 184, and less stiff “PDMS 50/50” was made of 50% SylGard 184 and 50% SylGard 527. Each was cured at 65 °C for 18 h. PDMS wafers were ~1 mm thick and 12 mm in diameter. Electrospinning on these substrates produced a layer of electrospun fibers that was irreversibly bound to the support, such that fiber-support adhesion was not considered to be a confounding variable.

#### 2.1.2. Suspended Fiber Mats

Suspended fiber mats (Figure 1B) were produced by fixing PS rings of varying inner diameter to the rotating mandrel with double sided tape. The rings were cut so that the outer diameter of the ring was 16 mm and the inner gap diameter was either 3 or 10 mm. After electrospinning, a free, suspended EFM area was produced inside the annular ring.

#### 2.1.3. Tension-Released Fiber Mats

Tension-released fiber mats (Figure 1C) were produced by electrospinning on non-stick aluminum foil attached to the rotating mandrel. EFM sections were carefully cut with a scalpel, removed from the foil, and fixed to PDMS annular rings (as above) with gap diameters of 10 mm using Sylastic (Dow Corning, Midland, MI, USA). This produced a layer of electrospun fibers irreversibly bound to the support.

### 2.2. PDMS Control Substrates

As a control for supported EFM studies, cell adhesion was also compared to bare PDMS wafers free of EFMs. PDMS was synthesized as above, then air plasma treated for 10 min without cracking the PDMS surface to render surfaces hydrophilic for cell adhesion [28].

### 2.3. Scanning Electron Microscopy

For examination of fiber alignment, each type of EFM construct was attached to an aluminum stub using carbon tape (Ted Pella, Inc., Redding, CA, USA), sputter coated with gold for 30 s (Model 3 Sputter Coater 91000, Pelco, Reading, CA, USA) and imaged using a scanning electron microscope (SEM) (Quanta 200 SEM, FEI Company, Hillsboro, OR, USA). Fast Fourier Transform (FFT) was used to quantitatively validate fiber alignment [29,30].

### 2.4. Mechanical Characterization

The mechanics of the EFM constructs were characterized by measuring the Young’s modulus and calculating the indentation moduli using finite element modeling (FEM).

#### 2.4.1. Elastic Stress Characterization

Tensile testing (RSA2, New Castle, DE, USA) was carried out on PDMS 100/0, PDMS 50/50, and PS supports as well as 50 µm and 200 µm thick EFMs. EFMs were oriented in the aligned fiber direction for testing. Each was cut to a standard dogbone shape and tested up to a strain of 10% at a strain rate of 0.6% per second. The linear portion of the stress–strain curve was used to determine the Young’s modulus of the samples.

#### 2.4.2. Finite Element Model Indentation Characterization

A Finite Element Model (FEM) was created using ABAQUS/CAE 6.13-1 software (Dassault Systèmes Simulia Corporation, Providence, RI, USA, 2013). This model featured a 10 µm diameter spherical indentation (E = 200 GPa, Poisson’s ratio = 0.3) impressed into each EFM by 5 µm. Based on EFM morphology, we assume rigid, non-slip contact with the support. In the model, EFMs were either fixed along the base and outside edge (supported fiber mats) or solely on the outside edge (suspended fiber mats) (shown in Supplementary Figure S1 and further described in the Supplementary Material). We tested suspended EFM gap diameters of 3 and 10 mm, with EFM thicknesses of 50, 100 and 200 µm. An axisymmetric model captured the geometry of the EFMs and spherical indentation. The EFMs were assumed to be elastic and isotropic with a Young’s modulus of 7.9 MPa and a Poisson’s ratio of 0.35. Isotropy was assumed as shear modulus for PCL electrospun fibers is not statistically significantly different in perpendicular directions [31]. The indentation modulus of each EFM was determined by the Hertzian-contact method, which is valid for ideal elastic materials under infinitesimal deformation [32]. The elastic modulus of the substrate, given a rigid spherical indenter, is based on the load-displacement curve, as in Equation (1).

(1) $$E=\sqrt{\frac{S^{3}{\left(1-\nu^{2}\right)}^{2}}{6\mathrm{RP}}},$$

where E is the elastic modulus of the substrate, ν is Poisson’s ratio of the substrate, R is the nominal radius of curvature of the indenter tip, P is the applied load, and S is the material stiffness (S = dP/dh) evaluated at P.

### 2.5. Cell Culture

Human glioblastoma U87 MG cells (ATCC) were used for all experiments because of their highly invasive nature. Cells were cultured at 37 °C in a 5% CO_2_ atmosphere in DMEM/F12 (Sigma-Aldrich, St. Louis, MO, USA) supplemented with 10% fetal bovine serum (Fisher Scientific, Hampton, NH, USA), 1% penicillin/streptomycin (Invitrogen, Carlsbad, CA, USA) and 1% MycoZap (Life Technologies, Carlsbad, CA, USA). Cells were fed 2–3 times a week and passaged at confluence prior to use.

### 2.6. Analysis of Cell Morphology on PDMS Substrates and PCL Electrospun Fiber Mats

Supported fiber mats were fixed to the bottom of a 12 well plate using Sylastic to prevent floating. Suspended or tension-released EFMs were first fixed to a PDMS ring to prevent the unsupported area from touching the bottom of the well. Then, the PDMS ring was fixed to the bottom of the plate using Sylastic. PDMS wafers and EFMs were sterilized by soaking with 70% ethanol under UV for 20 min, then rinsed twice with PBS and once with media. Materials were then inoculated in cell culture media for 30 min. Cells were stained with CellTracker Green (Invitrogen, Carlsbad, CA, USA), then seeded at 40 k per well. After 18 h, cells were imaged using reflectance microscopy (Olympus IX-2, Shinjuku, Tokyo, Japan) near the center of the fiber mat to avoid potential edge effects. Feret diameter is the largest distance between any two points of an object and was measured using Image J Image Analysis Software (v1.52n, Bethesda, MD, USA) freely available from the National Institutes of Health.

### 2.7. Statistical Analysis

All data was analyzed using JMP statistical analysis software (JMP Pro 14, Cary, NC, USA). Statistical differences in cell response were detected with ANOVA and Tukey–Kramer HSD tests. In all cases, *p* < 0.05 was considered statistically significant.

## 3. Results

### 3.1. Fiber Mat Characterization

**Figure 2:**
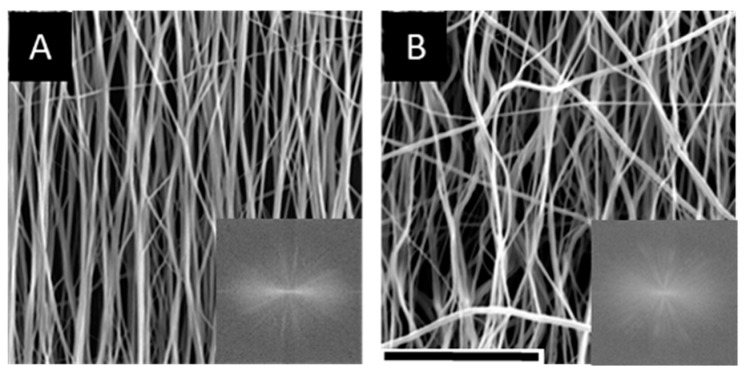
Representative scanning electron microscope (SEM) images of EFMs. Aligned (**A**) and tension-released (**B**) 100 µm thick EFMs showing differences in fiber morphology following release of internal tension. Insets: Fourier transform of the figure, indicating degree of alignment. Scale bar = 50 µm.

Correlating to standard EFM characterization methods, we first characterized EFM morphology and stiffness. Aligned EFMs were spun directly onto PS or PDMS to create supported (solid) or suspended (annulus) scaffolds (Figure 1A,B). Alternatively, EFMs were spun onto non-stick foil, cut and removed from foil to release internal tension arising from the electrospinning process, and fixed to a PDMS annulus to create tension-released scaffolds (Figure 1C). As-spun EFMs demonstrated excellent alignment (Figure 2A), some of which was preserved in the tension-released EFMs, though a clear non-linearity is introduced to the fibers (Figure 2B). In these representative EFM images, individual fiber diameter was consistent at 0.97 ± 0.04 µm and 1.00 ± 0.04 µm for 100 µm thick supported and tension-released EFMs, respectively (*n* = 127 total individual fibers). Fiber diameter distribution was normal (Gaussian) for PS-supported EFMs, but not for the tension-released EFMs (Supplementary Figure S2). Fiber densities in these EFMs were statistically different at 595.8 ± 17.6 mm^−1^ and 539.3 ± 11.7 mm^−1^ for 100 µm thick supported and tension-released EFMs, respectively (*p* = 0.0318, two-sided *t*-test). At the cellular level (~10 µm across), this is a difference between cells interacting with 5.96 fibers or 5.39 fibers. This may be negligible when considering that out-of-plane fibers were quantified, while cells largely interact with the top layer of fibers and do not penetrate the EFM. These fiber diameters and densities are very similar to our previously published results from the same electrospinning process [19].

Mechanical properties of EFMs were first characterized by calculating an FEM-generated indentation modulus (Table 1). The indentation modulus is based on an axisymmetric model simulating a rigid steel indenter impressing an EFM at its center. The indentation modulus for PS-supported EFMs was up to ~500×, or 8.233 MPa, higher than the indentation modulus for suspended EFMs of the same thickness. However, moduli for supported EFMs of different thicknesses were similar (e.g., 8.250 vs. 7.443 MPa). In contrast for suspended EFMs, the indentation modulus increased by ~50×, or 0.79 MPa, as the thickness of the suspended EFM increased from 50 to 200 μm at constant gap diameter (i.e., 10 mm). Finally, indentation modulus decreased by ~10×, or 0.145 MPa, as the gap diameter increased from 3 to 10 mm. Thus, the weight of each factor on indentation modulus from greatest to least was supported vs. suspended, EFM thickness (suspended), and gap diameter. Based on this model, supported presentations, increasing EFM thickness (suspended), and decreasing gap diameter all lead to a higher indentation modulus. Supported EFMs are generally stiffer than suspended EFMs. This suggests that decreasing gap diameter leads to a mechanical response more similar to a stiffer, supported EFM.

A uniaxial tensile test was then performed on the EFM support materials (i.e., PS and PDMS) and on the unsupported EFMs. The PDMS 100/0 was made of 100% SylGard 184 and PDMS 50/50 was mixed with 50% SylGard 527 to make it less stiff while keeping surface charge and chemistry constant [27]. As expected, PDMS 50/50 was less stiff, by about 1.5×, than PDMS 100/0 (Table 2). For EFMs with different thicknesses, the mean Young’s modulus increased with thickness, although results were not statistically significant. The 200 µm EFM was 1.4× stiffer than the 50 µm EFM (Table 2). These results are similar to indentation modulus calculations for supported EFMs (Table 1), which show that modulus is not a strong function of thickness. Thus, we conclude that EFM thickness does not play a strong role in the mechanical response as measured by Young’s modulus.

### 3.2. The Role of the Support in EFM Scaffolds

**Figure 3:**
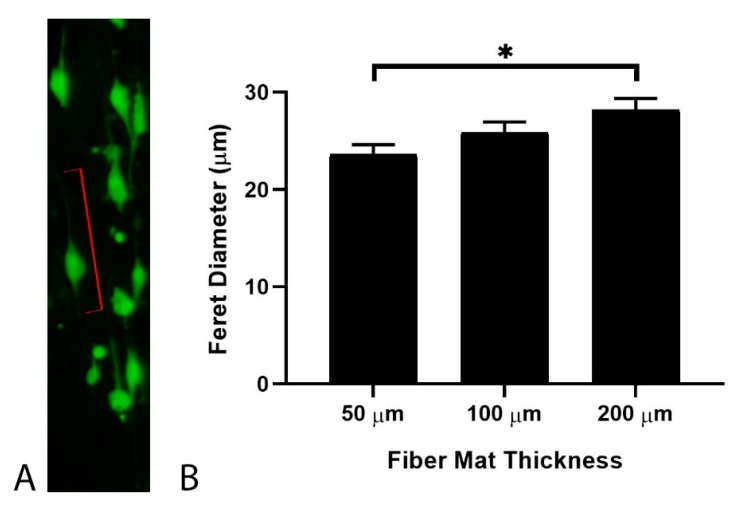
Glioblastoma cell Feret diameter (**A**) as a function of polystyrene (PS)-supported EFM thickness (**B**). Feret diameter is indicated for a representative cell (red lines). A total of 979 cells were analyzed across at least two independent experiments. Levels connected by a star (*) are statistically significant from each other (*p* < 0.05).

In our previous work [20], we showed that the mechanics of an underlying support material can influence the response of cells cultured at close proximity by inducing edge effects in an interfacial region. To investigate how such edge effects can potentially be utilized in EFM scaffolds, cells were grown on EFMs of varying thickness and with differing support materials (Figure 1A). Cell morphology was assessed by the Feret diameter (Figure 3A, red lines), which is the longest distance between any two points of an object. We have previously shown that cells exhibit polarized morphologies on aligned EFMs and that Feret diameter correlates with migration speed for glioblastoma cells [19], the model system employed here. Increasing EFM thickness from 50 to 200 μm led to a statistically significant increase of ~19% in cell Feret diameter for PS-supported EFMs (Figure 3B). Thus, although a statistically significant difference in Young’s modulus could not be detected, statistically significant cell responses were still observed.

**Figure 4:**
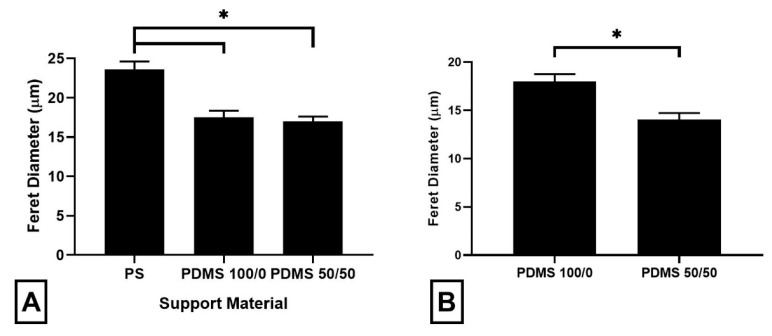
Glioblastoma cell Feret diameter in response to culture on 50 µm EFMs supported on substrates of declining stiffness (**A**) and on polydimethylsiloxane (PDMS) supports of declining stiffness with no EFM present (**B**). A total of 1623 cells were analyzed across at least two independent experiments. Levels connected by a star (*) are statistically significant.

Cells were also cultured on EFMs at a thickness of 50 µm using support materials of decreasing Young’s modulus (i.e., PS, PDMS 100/0 and PDMS 50/50) (Figure 4A). PS was selected because it exhibits a Young’s modulus several orders of magnitude higher than that of the EFMs employed (i.e., ~2200 vs. ~15 MPa for EFMs), similar to our previous work examining Matrigel (e.g., 1 kPa) supported on glass (e.g., GPa) [20]. For comparison, two types of softer PDMS were employed; both with Young’s moduli lower than that of the EFMs (3.13 and 1.29 MPa, respectively vs. 15.00 MPa for EFMs). We observed a ~26% decline in Feret diameter between the PS and the PDMS 50/50-supported 50 µm EFMs. In contrast, no difference in Feret diameter was detected between the two PDMS-supported models. However, when the cells were cultured directly on PDMS 100/0 and PDMS 50/50 support materials without an EFM, a significant difference in Feret diameter was seen (Figure 4B). This suggests that the mechanical response of the EFM scaffold is a combination of the support material and the EFM itself. Thus, tuning the mechanobiological response of cells to EFM scaffolds may be achieved either by altering the EFM thickness or by altering the stiffness of the underlying support material and that cells are able to sense the stiffer material in the interfacial region of composites.

### 3.3. The Role of Suspension in EFM Scaffolds

**Figure 5:**
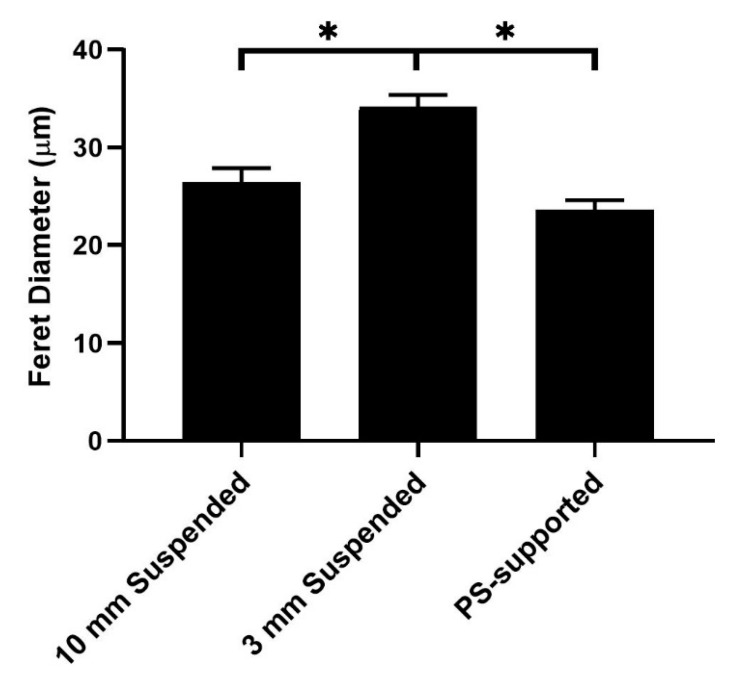
Glioblastoma cell Feret diameter on supported (PS) and suspended (10 mm, 3 mm) 50 µm EFMs. Scaffolds are arranged left to right by increasing indentation modulus. A total of 935 cells were analyzed across at least two independent experiments. Levels connected by a star (*) are statistically significant from each other (*p* < 0.05).

To further elucidate how mechanical nuances can influence cell behavior, we investigated EFMs lacking support in a central region, i.e., EFMs suspended across an annulus (Figure 1B). This architecture resembles tissue engineered constructs in which a support material may be missing or only partially present [12,13], such as artificial trachea constructs. PS disks were used to suspend EFMs of different thicknesses (i.e., 50, 100 and 200 µm) over two inner gap diameters (i.e., 3 and 10 mm). Compared to 50 µm PS-supported EFMS, cells cultured on 50 µm EFMs supported across a 10 mm annular gap displayed Feret diameters similar in magnitude, but cells on the 3 mm gap scaffold had a significantly higher mean Feret diameter than either support (Figure 5). According to FEM, the 3 mm gap scaffold would have an indentation modulus between that of the 10 mm gap scaffold and the PS support. 

**Figure 6:**
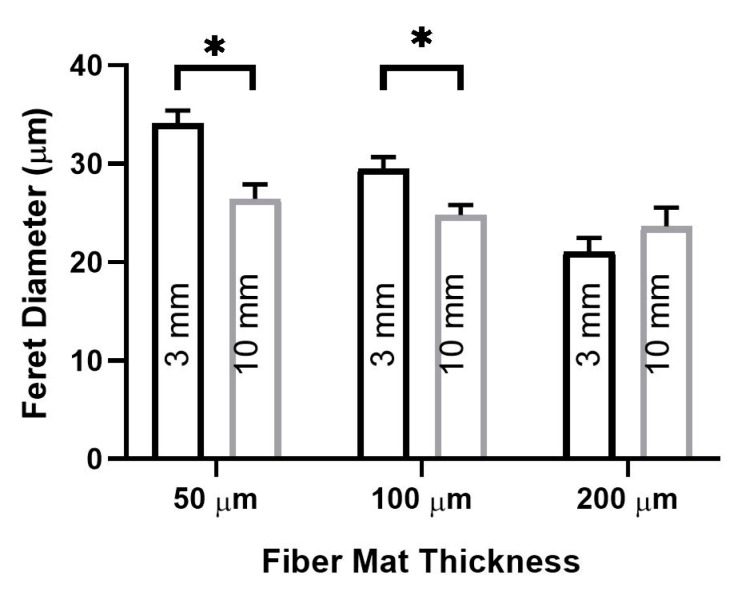
Glioblastoma cell Feret diameter measured on supports with 3 and 10 mm diameter annular gaps supporting EFMs of different thicknesses. A total of 1097 cells were analyzed across at least two independent experiments. Levels connected by a star (*) are statistically significant from each other (*p* < 0.05).

To gain additional information on these responses, we investigated the effect of EFM thickness coupled to annular diameter (Figure 6). As EFM thickness increased from 50 to 200 μm, Feret diameter decreased by ~37% for a fixed 3 mm gap diameter. At the larger 10 mm gap, this decrease was much less pronounced (~18%). In addition, there was a statistically significant difference in Feret diameter as a function of gap diameter for the 50 μm thick and 100 μm thick EFMs. This difference was abrogated at the 200 µm EFM thickness. Thus, increasing gap diameter correlates with a decreased indentation modulus and an observed decrease in Feret diameter. Interestingly, increased EFM thickness correlates with increased indentation moduli, but in this case caused a decreased Feret diameter. This highlights the mechanical complexity of EFM scaffolds, especially in the more nuanced scenario of a suspended EFM. These results are likely influenced by the role of curvature, which could be pronounced at the length scale of a cell. Alternatively, these responses may reflect internal residual tension that is introduced by deformation of the EFM in the central region.

### 3.4. The Role of Tension in EFM Scaffolds

**Figure 7:**
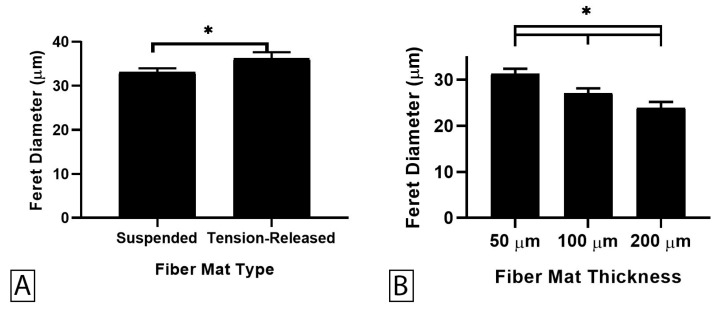
Average cell Feret diameter observed on tension-released and suspended EFM constructs, pooled data across all EFM thicknesses (**A**) and between tension-released mats at different fiber mat thicknesses (**B**). A total of 1651 cells were analyzed across at least two independent experiments. Levels connected by a star (*) are statistically significant from each other (*p* < 0.05).

To further explore the role of internal tension, suspended EFMs were modified to reduce internal residual tension by electrospinning onto non-stick aluminum foil, cutting the fibers and removing them from the foil, and fixing free-standing EFMs to PDMS annular rings with an inner gap diameter of 10 mm (Figure 1C). These “tension-released” EFMs produced a statistically significant increase of 9.7% in Feret diameter compared to suspended EFM without released tension at the same gap diameter (Figure 7A). In addition, increasing EFM thickness led to lower Feret diameter (Figure 7B). This is the same trend observed for suspended EFMs. In this case, we have empirically reduced the stiffness of the EFMs by releasing internal tension, yet EFM thickness remains inversely correlated with Feret diameter in contrast to results on supported EFMs (Figure 3B). Taken together, this data suggests that the mechanical environment of EFM scaffolds is complex and that cells may react to this environment in different ways on depending on the stiffness and presentation of the support.

## 4. Discussion

This work explores the impact of EFM mechanical parameters, beyond Young’s modulus, on cell morphology as measured by the Feret diameter. In particular, the effect of the mechanics of the support, the presence or absence of the support, inherent tension in EFMs and EFM thickness were explored. For supported EFMs, changing the stiffness of the support material underlying the EFM caused a large change in cell morphology, but only if the support was much stiffer than the EFM (Figure 4A). This is not because cells could not sense differences in stiffness between the underlying substrates; indeed, Feret diameter was different when grown directly on softer flat PDMS supports without an EFM (Figure 4B). However, when the much stiffer EFM was added, these differences were abrogated. Although this could result from topography differences, EFMs are fibrous whereas PDMS supports are relatively smooth and flat, others have shown that cells can “feel” the underlying substrate when cultured on thin hydrogels. Experiments in which cells spread more on thin gels than on thicker gels demonstrate this interplay of support substrates [33]. 

We are the first to demonstrate such edge effects in EFMs. Buxboim et al. described a “threshold matrix thickness,” the length scale at which cells respond not only to the stiffness of the matrix, but also to the rigidity of the underlying support for hydrogel models [21]. However, these length scales were on the order of 10–20 µm compared to our observed responses on 50–200 µm thick EFMs. Cells may be able to “feel” further when grown on EFMs than when grown on hydrogels because of the orders of magnitude difference in length scales of individual fibers. Electrospun fiber length can range upwards of 35–50 cm [34], whereas collagen fibers in hydrogels are only 0.5–3 µm long [35]. Other factors aside (e.g., fiber strength, fiber interconnectivity, etc.), this difference in length scale may lead to differences in how well the matrix transmits tension. This likely permits stress to be transferred over a larger distance. 

Suspended EFMs produced perhaps the most intriguing results. From a design perspective, the 50 µm thick, 3 mm gap EFM scaffolds yielded a key finding; these substrates elicited the highest mean Feret diameter of all (Figure 5). Cells on a thin EFM suspended over a small gap spread more than cells on a thin EFM fixed to a rigid support with a higher indentation modulus (Table 1). In general, cells on stiffer substrates demonstrate higher contractility and spreading [36], i.e., Feret diameter. Engler et al. demonstrated cells grown on hydrogels show a bell curve-shaped response to ligand density, though ligand density and substrate stiffness are highly difficult to decouple in gels [37]. We have previously shown that this bell curve-shaped response also exists in EFMs for cell mechanics when ligand density and stiffness were decoupled through the use of core-shell electrospinning [19]. Here, we have extended upon that work by decoupling material properties from more complex composite and structural mechanics by suspending fiber mats across gaps of different diameter without changing surface ligand density or Young’s modulus. We also interrogated focal adhesion kinase (FAK) expression through Western blotting and found no significant differences in expression (Supplementary Figure S3). Although FAK phosphorylation could play a role, this suggests that this mechanosensing mechanism may not be mediated by classical focal adhesion kinase machinery, which we have previously implicated in EFM mechanotransduction [19]. These data help to illustrate the complex interplay of substrate mechanics, cell adhesion molecules, and mechanosensing.

For suspended EFMs, changing gap diameter caused significant changes in cell morphology (Figure 6). Releasing the internal tension in EFMs also altered mean Feret diameter (Figure 7A). These results highlight the interplay of substrate mechanics and mechanosensing beyond Young’s modulus. It is generally believed that cells spread more on stiffer substrates [36]. Our investigation into the effect of support material stiffness (Figure 4) generally corroborates this belief; Feret diameter correlated positively with stiffness, as measured by Young’s modulus. However, these results were inverted with EFM thickness for both supported and suspended EFM presentations (Figure 3B and Figure 6). Feret diameter inversely correlated with stiffness, as measured by indentation modulus. In supported and suspended EFMs, increasing EFM thickness had opposite effects on indentation modulus. Increasing supported EFM thickness results in only a slight decrease in indentation modulus; however, increasing suspended EFM thickness resulted in ~50× increase in indentation modulus (Table 1). For supported scaffolds, a given cell at the center of the EFM is only hundreds of microns from the rigid support (i.e., one EFM thickness away), whereas for suspended scaffolds the rigid support is millimeters away. This likely affects how the cell feels mechanics between these models. Suspending EFMs may also cause an impactful change in the curvature of the EFM. These complexities that arise from inverted cell response to stiffness and suspending EFMs are reflected in part by the bell curve-shaped response to indentation modulus when comparing suspended and supported presentations (Figure 5). Further experiments are warranted to decouple these variables to explain these differences. 

In addition, improved characterization employing more advanced FEM models that consider individual fibers over different length scales or empirical data that recapitulates cell contractility would yield helpful insights. We employ an FEM-generated indentation modulus and an experimentally derived Young’s modulus. Neither completely describes the results seen herein. Young’s modulus is especially poorly-suited to provide insights because the most challenging data arise specifically from changes in structural mechanics, not changes in the materials themselves. Thus, systems have identical Young’s moduli.

These data highlight a gap in understanding between cell sensing and response to the mechanical environment. Further, the large differences generated by small, nuanced changes in the mechanical environment, for example a ~37% change in morphology with a change in EFM thickness (Figure 6), suggest caution in interpreting data from mechanical studies that may lack scientific rigor. In addition, these data suggest new variables (i.e., support material, suspension diameter) that can be tuned to alter material mechanobiology effects. The field of biomaterials has historically progressed from early generation scaffolds that are bioinert to later generations that are bioactive or bioresorbable. For example, earlier generation bioinert alumina vs. more recent bioresorbable tri-calcium phosphate dental implants [38]. Whereas it is acceptable to “avoid” biological complexity early, this complexity also provides powerful opportunities to improve patient outcomes. Impending generations of biomedical scaffolds will need to harness the powerful, yet relatively untapped realm of nuanced mechanobiology.

## Figures and Tables

**Table 1:** Indentation modulus (MPa) for electrospun fiber mat constructs.

	50 µm	100 µm	200 µm
Supported, PS	8.250	7.814	7.443
Suspended, 3 mm gap diameter	0.162	1.069	3.932
Suspended, 10 mm gap diameter	0.017	0.118	0.807

Indentation modulus generated by finite element modeling.

**Table 2:** Young’s modulus of electrospun fiber mats and support materials.

Material	Young’s Modulus (MPa)
50 µm EFM	15.00 ± 1.01
200 µm EFM	20.76 ± 3.37
PS	2160.63 ± 51.35
PDMS 50/50	1.29 ± 0.10
PDMS 100/0	3.13 ± 0.35

Data are displayed as mean ± standard error.

## Supplementary Materials

The following are available online at https://www.mdpi.com/2079-4991/9/4/644/s1, Figure S1: Boundary conditions and interactions for indentation FE models, Figure S2: Individual fiber diameter distributions. Frequency distributions for PS-supported (A) and tension-released (B) EFMs, Figure S3: Focal adhesion kinase expression.
