# Supplementary material for: Effect of Electrospun Fiber Mat Thickness and Support Method on Cell Morphology
Source: Nanomaterials (Basel). 2019 Apr 20;9(4):644. doi: 10.3390/nano9040644 (PMC6523829; doi:10.3390/nano9040644)
Supplement: Supplementary file 1 [file nanomaterials-09-00644-s001.pdf]

Supporting Information

Effect of Electrospun Fiber Mat Thickness and Support Method on Cell Morphology

Mark A. Calhoun [1], Sadiyah Sabah Chowdhury [2], Mark Tyler Nelson [1], John J. Lannutti [3], Rebecca B. Dupaix [2] and Jessica O. Winter [1,4,*]

[1] Department of Biomedical Engineering, The Ohio State University, Columbus, OH 43210, USA; Calhoun.89@osu.edu (M.A.C.); Nelsonmt05@gmail.com (M.T.N.)
[2] Department of Mechanical and Aerospace Engineering, The Ohio State University, Columbus, OH 43210, USA; Chowdhury.68@osu.edu (S.S.C.); Dupaix.1@osu.edu (R.B.D.)
[3] Department of Materials Science and Engineering, The Ohio State University, Columbus, OH 43210, USA; Lannutti.1@osu.edu
[4] William G. Lowrie Department of Chemical and Biomolecular Engineering, The Ohio State University, 453 CBEC, 151 W. Woodruff Ave., Columbus, OH 43210, USA
* Correspondence: winter.63@osu.edu; Tel.: +1-614-247-7668

Figure S1. Boundary conditions and interactions for the indentation FE models. (A) Suspended boundary condition, where only the outer edges are fixed and (B) supported boundary condition, where the base and the outer edges are fixed.

Figure S2. Individual fiber diameter distribution. Frequency distributions for PS-supported (A) and tension-released (B) EFMs.

Figure S3. Focal adhesion kinase expression. Western blots (A) and densitometry for each comparison (B–D).

1. Supplemental Methods

1.1 Finite Element Modeling

Two different boundary conditions were applied in model simulations: a suspended boundary condition (Figure S1A) and a supported boundary condition (Figure S1B). The condition of symmetry was applied to both boundary conditions. Since the indenter is an analytical rigid material, a reference point was assigned to indicate the rigid body reference point ("RP-1", Figure S1). Constraints or motion applied to RP-1 were applied to the entire indenter. The interaction between the indenter and the fiber mat was modeled as a surface to surface contact with finite sliding where the indenter acts as the master surface and the fiber mat acts as the slave. The indenter and the fiber mat were meshed using quadratic elements. A partitioned mesh was created in the fiber mat with a finer mesh close to the contact region ("partition", Figure S1) to capture the stresses, strains, and deformations with reasonable accuracy.

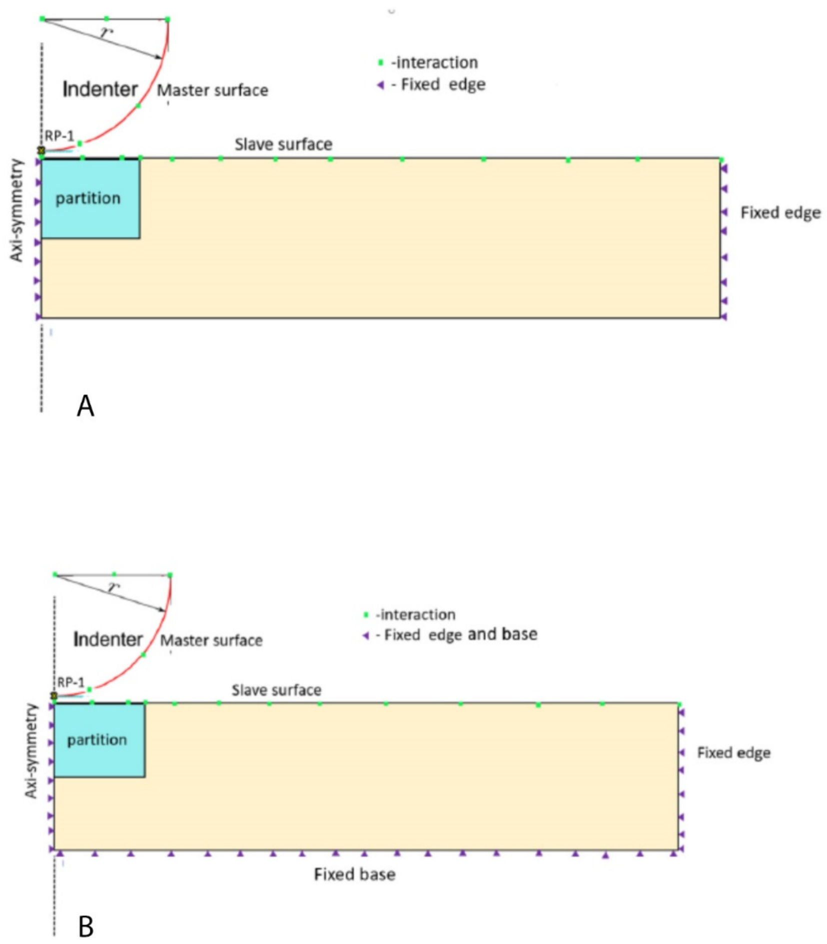

Figure S1. Boundary conditions and interactions for indentation FE models. Suspended boundary condition (A), where only the outer edges are fixed, and supported boundary condition (B), where the base and the outer edges are fixed.

1.2 Analysis of focal adhesion kinase signaling using western blotting

Suspended EFM (50 and 200 µm, 3 and 10 mm gap diameter) and 50 µm thick supported EFM constructs were sterilized as previously described. Cells were seeded at a density of 2 × 10^5 cells per well and incubated for ~ 18 h. Fiber mat constructs were rinsed repeatedly with loading/lysis buffer consisting of pH 6.8, 50 mM Tris-HCl, 1% SDS, 1 mM EDTA, 5% glycerol, and 1× Halt protease and phosphatase inhibitor (ThermoFisher Scientific 78442). Total cell lysates were resolved on an SDS-PAGE gel and transferred to a polyvinylidene fluoride (PVDF) membrane. Blots were labeled for FAK (Cell Signaling 3285) and Actin (Sigma A5441), which served as a loading control. Proteins were visualized using Cy3 or Cy5 ECL-Plex secondary antibodies and a Typhoon scanner. Densitometry was measured in ImageJ by calculating the background-subtracted relative density of the protein bands relative to the actin loading control, and then normalizing each condition to the suspended, 3 mm gap diameter, 50 µm thick condition.

2. Supplemental Results

2.1 Fiber diameter distribution

Individual fiber diameters were measured for PS-supported and tension-released EFMs (Supplementary Figure 2). Mean fiber diameters were similar at 0.97 ± 0.04 µm and 1.00 ± 0.04 µm for 100 µm thick supported and tension-released EFMs, respectively. According to the Shapiro-Wilk test of normality, the PS-supported fibers were normally distributed ($p = 0.2646$), but the tension-released fibers were not ($p < 0.0001$).

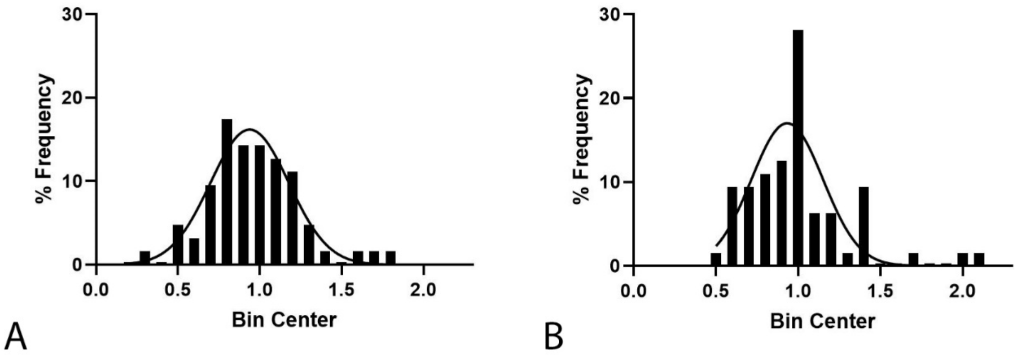

Figure S2. Individual fiber diameter distribution. Frequency distributions for PS-supported (A) and tension-released (B) EFMs.

2.2 Focal Adhesion kinase signaling

To evaluate correlation of Feret diameter changes to potential cell signaling pathways, expression of FAK was measured for cells cultured on supported and suspended EFM constructs (Figure S2A). The role of FAK in focal adhesions is two-fold, helping to transmit traction to the extracellular matrix and transducing extracellular inputs into the appropriate signal transduction pathways [1]. FAK has also been implicated in glioma migration [2,3], which we have previously shown correlates with Feret diameter in glioma cells on aligned EFMs [4]. However, there were no statistically significant changes in FAK expression in our study (One-way ANOVA, $p = 0.0964$). Although it is possible that phosphorylation of FAK is different, these results suggest that the mechanosensing signal transduction cascade are independent of FAK expression, and possibly classical FAK machinery.

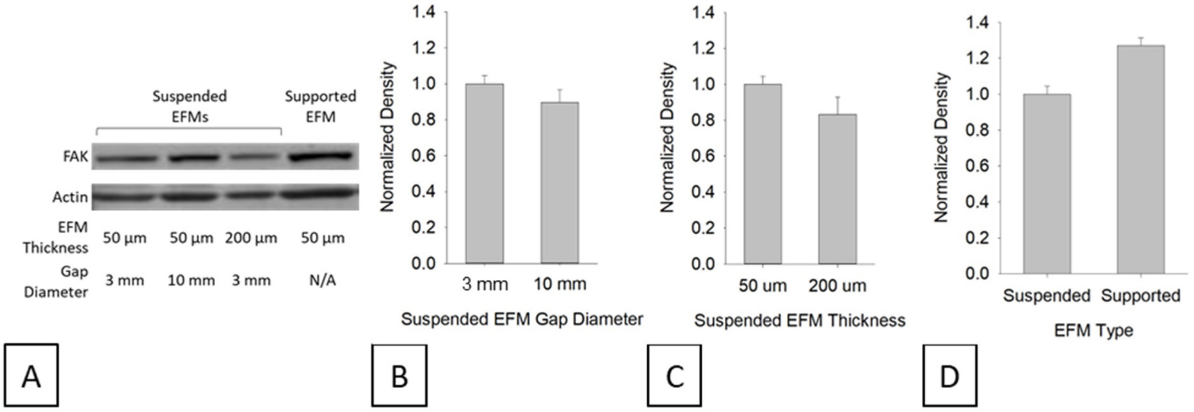

Figure S3. Focal adhesion kinase expression. Western blots (A) and densitometry for each comparison (B–D).

3. Supplemental References

1. Mitra, S.K.; Hanson, D.A.; Schlaepfer, D.D. Focal adhesion kinase: in command and control of cell motility. *Nature reviews. Molecular cell biology* **2005**, *6*, 56-68, doi:10.1038/nrm1549.
2. Cox, B.D.; Natarajan, M.; Stettner, M.R. New concepts regarding focal adhesion kinase promotion of cell migration and proliferation. *Journal of cellular* **2006**, 10.1002/jcb.20956, doi:10.1002/jcb.20956.
3. Natarajan, M.; Hecker, T.P.; Gladson, C.L. FAK Signaling in Anaplastic Astro cytoma and Glioblastoma Tumors. *The Cancer Journal* **2003**.
4. Rao, S.S.; Nelson, M.T.; Xue, R.; DeJesus, J.K.; Viapiano, M.S.; Lannutti, J.J.; Sarkar, A.; Winter, J.O. Mimicking white matter tract topography using core–shell electrospun nanofibers to examine migration of malignant brain tumors. *Biomaterials* **2013**, *34*, 5181-5190, doi:10.1016/j.biomaterials.2013.03.069.
